# All for One Health and One Health for All: Considerations for Successful Citizen Science Projects Conducting Vector Surveillance from Animal Hosts

**DOI:** 10.3390/insects13060492

**Published:** 2022-05-24

**Authors:** Karen C. Poh, Jesse R. Evans, Michael J. Skvarla, Erika T. Machtinger

**Affiliations:** 1 Department of Entomology, Penn State University, University Park, PA 16802, USA; jesserayevans@psu.edu (J.R.E.); mxs1578@psu.edu (M.J.S.); etm10@psu.edu (E.T.M.); 2 USDA-ARS Animal Disease Research Unit, Pullman, WA 99164, USA

**Keywords:** citizen science, active surveillance, passive surveillance, vector surveillance, vector-borne disease, domestic animals, wildlife, United States, Canada

## Abstract

**Simple Summary:**

Vector-borne diseases are often zoonotic and so a One Health approach must be employed in order to investigate and control them. Therefore, surveillance of arthropod vectors and pathogens among animal populations should complement human disease surveillance. Since traditional surveillance methods to collect arthropod vectors and conduct pathogen testing from animals can be challenging, data collection can be supplemented with citizen science approaches, where the general public is actively involved in collecting animals and/or samples. In this review, we discuss considerations for researchers to create a successful vector surveillance program using citizen science approaches with different stakeholders who own, have interests in, or work with animals.

**Abstract:**

Many vector-borne diseases that affect humans are zoonotic, often involving some animal host amplifying the pathogen and infecting an arthropod vector, followed by pathogen spillover into the human population via the bite of the infected vector. As urbanization, globalization, travel, and trade continue to increase, so does the risk posed by vector-borne diseases and spillover events. With the introduction of new vectors and potential pathogens as well as range expansions of native vectors, it is vital to conduct vector and vector-borne disease surveillance. Traditional surveillance methods can be time-consuming and labor-intensive, especially when surveillance involves sampling from animals. In order to monitor for potential vector-borne disease threats, researchers have turned to the public to help with data collection. To address vector-borne disease and animal conservation needs, we conducted a literature review of studies from the United States and Canada utilizing citizen science efforts to collect arthropods of public health and veterinary interest from animals. We identified common stakeholder groups, the types of surveillance that are common with each group, and the literature gaps on understudied vectors and populations. From this review, we synthesized considerations for future research projects involving citizen scientist collection of arthropods that affect humans and animals.

## 1. Introduction

Vector-borne diseases (VBDs) caused by pathogens that are transmitted by arthropod vectors are a global concern to human and animal health. In the United States (U.S.) and U.S. territories, human cases of VBDs have tripled since 2004, and nine human VBDs were first reported between 2004 and 2016 [1]. Many of these VBDs are zoonotic, or transmissible among animals and humans, and some circulate only in animal populations, where they cause extreme illness or death. The cost of treatment or loss of animals, including livestock, from VBDs is an economic and emotional burden and, in some cases, these VBDs are notifiable diseases that can impact international trade of animals [2]. Because many VBDs are considered zoonotic, there is a need for a One Health approach to better understand vectors and potential vectors, their hosts, and the pathogens they carry to reduce the global impact of VBDs in humans and animals. Taking the One Health approach and understanding the connection between human and animal health will ultimately lead to healthier communities. To do so, vector and VBD surveillance should take place in both human and animal populations, but many traditional surveillance methods to collect data are often constrained by time, resources, and labor.

The increased need for data on vectors in North America has led to a subsequent increase in the number of citizen science projects focused on vector biology and control. Citizen science projects involve public volunteers that contribute to a research program by collecting and/or analyzing data. These projects are overseen by a research team of researchers and the concepts are adapted and applied to diverse disciplines (reviewed in [3,4]). These types of coordinated projects have been successful at advancing scientific knowledge with the acquisition of large datasets that may have otherwise been unattainable because of limitations of time and resources [5]. Citizen science projects have generated data on arthropod vectors including human–vector encounters (e.g., bites), species distributions, phenology, efficacy of control methods [6], and detection of invasive species [7], and these data have been used to inform control programs, increase social responsibility of vector control [8], and facilitate policy changes [7].

The design of citizen science projects can vary significantly based on temporal and spatial parameters and data can be collected in different ways. Often, data collected using citizen science are considered passively collected from the perspective of the research team and have been referred to as such in other topic reviews because of the minimal effort needed to receive samples after the study is initiated. Traditionally, data analyses can be constrained to data collected and provided to the research team by citizen scientists involved in the study, but data are not always exclusively collected by the citizen scientists. Project design and data analysis can instead take an integrative approach, whereby the citizen scientists and research team combine efforts to collect data that are later analyzed by the researchers.

Many actual or potential biological vectors are associated with animal hosts. Working with animals in research to study vectors can be expensive and may include regulatory challenges and permitting. In addition, sample sizes are often limited by sampling effort, which reduces the robustness of the conclusions. However, projects involving animals can be well-suited to engaging citizen scientists as the public is frequently in contact with animals personally and/or professionally. For example, it is estimated that approximately 70% of households in the U.S. [9] and 58% of households in Canada [10] have at least one companion animal. Others may keep horses, livestock, and other traditional “farm” animals recreationally. Recent reports found that 11.5 million Americans [11] and 1.3 million Canadians [12] engage in hunting and trapping activities and therefore may have contact with various wildlife and game species. Other members of the public may have professional access to animals, including, but not limited to, veterinarians, livestock, and poultry farm owners or workers, game wardens, animal control officers, and shelter or rehabilitation managers or volunteers. These associations provide opportunities for vector studies by citizen scientists on many species of wild and domestic animals that would not otherwise be achieved by a single research group and can produce data that can be used to better understand vector ecology, protect human and animal health, and improve or develop novel control methods.

While there are numerous opportunities when conducting vector studies using involving citizen scientists, there are also communication, logistical, participation, and methodological challenges associated with different research objectives and stakeholder groups. Therefore, the purpose of this review is to synthesize the details and implications of citizen science projects based in the U.S. and Canada that have specifically incorporated animals to study vectors and VBDs, and discuss the methodology, benefits, challenges, and stakeholder considerations associated with professional companion animal access, personal companion animal access, professional wildlife access, and recreational wildlife access. Subsequently, gaps and considerations for future citizen science studies are suggested.

## 2. Professional Companion Animal Access

People who handle or work with animals as part of their occupation have previously shown success as reliable data sources for arthropod vectors. This group of citizen scientists includes veterinarians, domestic animal control or animal shelter personnel, humane societies, and those working at livestock markets or farms. Many studies targeting this stakeholder group have used an exclusive citizen-led approach, where citizen scientists collected ectoparasites or photographs of ectoparasites from domestic animals and submitted them to researchers.

### 2.1. Surveillance and Data Collection Methodology

Ectoparasite collection was typically conducted when the citizen scientists interacted with the animal as part of their clientele (e.g., during a veterinary checkup or bringing stray animals into a shelter). To assist with vector collections, citizen scientists were either sent collection kits and materials [13,14,15,16,17] or exclusively given instructions via email, print material, or project website [18,19,20] on procedures to collect, preserve, and send specimens to researchers. When considering whether to send collection kits to citizen scientists, researchers should consider the cost to mail and receive the kits, whether special chemicals for specimen or pathogen preservation are required, and how differences in preservation or submission could affect the specimen or pathogen status. Skvarla et al. [21] reviewed preservatives in the context of terrestrial pitfall traps but many of the issues are applicable to arthropod preservation regardless of the specific application.

Materials for the collection kit, postage to send the kit, and postage to send the kits back to the research institution are factors that should be considered when determining cost. If a project involves a large sample size, then postage costs of sending and returning kits may be prohibitive. For example, Evans et al. [22] created collection kits which were light in materials (three microcentrifuge tubes, a pair of forceps, instructions, and datasheets) and cost approximately $3 per kit; however, the total cost of postage stamps was approximately $7.50 to send and receive the kits. Material costs may be reduced by purchasing in bulk or using less expensive alternatives, e.g., alcohol-based hand sanitizer instead of ethanol as a preservative [23].

Another method of collection kit distribution is sending or dropping off kits at designated locations where specific groups frequent, such as a veterinary clinic or hospital, livestock market location, or an Extension office [24]. While not a location they may frequent, those who work with companion animals can also pick up and drop off kits at the research institution housing the project. Regardless, having a pickup and drop-off location for collection kits has the benefit of eliminating postage costs, but may limit the study population to the areas immediately surrounding the pick-up/drop off location. If only instructions are sent, then postage costs are greatly reduced or eliminated if the instructions are sent digitally via an email or shared on a website. However, this places the financial cost of participation on the citizen scientists rather than the researchers and may decrease the number of people who participate in the study.

If no materials are given to citizen scientists, participants may still participate and collect specimens, depending on the ease of collection, preservation method required, and availability of containers to hold the specimen in transit. For example, if a veterinary clinic has a high patient load, they may not necessarily have the personnel or capacity to create their own preservation chambers for the ectoparasites. While different preservation chemicals (ethanol, hand sanitizer, etc.) and containers are widely available and accessible to many populations, biases in pathogenic data interpretation could be introduced, especially for viral pathogens which can break down during storage in ethanol [25,26]. Conversely, those who work in animal care to test and detect pathogens in animals may already have access to pathogen stabilization reagents that other occupations or other types of citizen scientists might not have access, which can lead to more inconsistencies in data interpretation. Therefore, if the collection kits require specific materials that may not be widely available to the targeted population, then the research team should consider creating the collection kits and identifying distribution methods that fit best with their budget and study objectives.

Given that physical specimen collection and submission can be costly to researchers and/or citizen scientists, another option to collect data points include the use of citizen-submitted images through a mobile device application (“app”) or website, where photos can be used for vector surveillance. The use of digital technology and big data for vector surveillance in human and animal populations has been increasing throughout Australia, Europe, and North America [7,27,28,29,30,31,32,33,34], however, only one study used digital technology to conduct vector surveillance among animals from people who have professional companion animal access [35]. The study evaluated the feasibility of images submitted by veterinarians for *Ixodes scapularis* surveillance in Quebec, Canada. The authors found a high percentage of correctly identified ticks amongst the images that were deemed acceptable for identification, reinforcing the feasibility of using images submitted by the public to conduct tick surveillance. Given that photo quality was important for identification, research leaders provided guidelines to train veterinarians on taking adequate photos [35]. To our knowledge, the use of citizen-submitted photos from this stakeholder group and for other vectors has not been conducted for the U.S.

### 2.2. Benefits

Collaborating with citizen scientists who have professional companion animal access has many benefits. One major reason to work with this group is that a passive surveillance system can be used to detect and track ectoparasites and VBDs that can affect pets/domestic animals, even over extended periods of time [17,18,36,37,38]. This is especially useful when considering invasive vectors that are introduced into new geographic regions. For example, Duncan et al. [13] and Trout-Fryxell et al. [15] relied on communication and action of this citizen scientist group to detect and track the spread of the invasive Asian long-horned tick (*Hamephysalis longicornis*), which was first reported in the U.S. in 2017 [39]. A similar program was also conducted in Michigan with *I. scapularis* and the causative agent of Lyme disease *Borrelia burgdorferi*, where citizen scientists, including those with professional access to companion animals, were asked to submit samples to develop a comprehensive distribution of the tick and bacterium throughout the state [40]. Similarly, the spread of *I. scapularis* into Ontario, Canada, relied on samples submitted from veterinarians to determine if *I. scapularis* and *B. burgdorferi* were widespread throughout the province, where the bacterium was still considered rare at the time [41]. Assuming those who work with animals have enough time and personnel to conduct surveillance, and if ectoparasite collection is relatively quick and easy, it is possible to track vector spread and prevalence via a long-standing passive surveillance system with this stakeholder population.

Because many pets and domestic animals are in close contact with this group of citizen scientists, researchers can determine the risk of ectoparasite bites to pets as well as human risk of ectoparasite bites and VBD spillover [17,20,42,43]. A major component of citizen science is to engage with the stakeholder group in order to share or develop ideas and research questions that are relevant to the audience and to listen to their concerns to prioritize research. Because of the increased risk of ectoparasite bites and VBD transmission to those who work with pets, there is also a need to develop targeted messaging for this specific population, which can in turn increase participation in the study. Furthermore, communicating research findings with stakeholders will reinforce open lines of communication for future collaborative efforts. By having this flow of communication and public engagement between researchers and stakeholders, trust and effective information dissemination can materialize [44,45].

### 2.3. Challenges

While there are major advantages to working with animal caretakers, there are also challenges to working with them. The first challenge is finding, enrolling, and retaining these citizen scientists to actively and consistently search all animals they encounter. Much of the participation from this citizen scientist group will largely rely on the personnel available to search, identify, and collect samples regularly. For example, if a clinic has a large patient population or workload, then qualified personnel may not always have the time available to collect samples. This could lead to gaps in datasets or inconsistencies in effort to collect data, which can ultimately lead to incorrect data interpretation and conclusions.

Sample sizes and scope of hosts and ectoparasites may also be limited by working with this group. Depending on the geographic location, researchers may not get as large of a sample size as compared to a broader audience. For instance, government jurisdiction may limit only one animal control unit or shelter within a city or county, which may constrain the sample size and coverage of sampled animals within the location of interest. In addition, the animals of interest are limited to those that are seen by those who have professional companion animal access (i.e., domestic pets, livestock, poultry, etc.). Depending on the focus of the clinic, shelter, or market/farm, exotic animals may be excluded from the study. The study may also be limited to ectoparasites that are found on these animals at high enough population densities for animal care takers to find, which may limit the detection of recently introduced or otherwise rare ectoparasites. These factors should be considered when deciding on study objectives or interpreting results involving professionals with companion animal access.

### 2.4. Stakeholder Considerations

There are certain considerations when deciding to work with people whose occupations involve working with pets and other domestic animals. First, researchers should consider the type of ectoparasite that will be collected, specifically pertaining to the collection method and the stakeholders’ familiarity with the ectoparasite(s). These two considerations will affect the availability of personnel to consistently check animals and collect ectoparasites as well as determine if trainings are required, respectively. Ideally, the arthropod should be easy to find and collect from the animal’s body and should not require excessive time or materials to collect, label, and store. Therefore, insects that fly or arthropods that readily leave their hosts post-feeding and are difficult to find without a microscope are not ideal for conducting citizen science research projects with this stakeholder group. Providing the necessary materials to search and collect arthropods of interest, especially if the study requires special equipment or chemicals, may also help with participation.

Familiarity of the ectoparasite is also important to consider when collaborating with those who work with pets. If animal care takers are unfamiliar with the ectoparasite and its biology, they could miss it during the animal inspection, send the incorrect types of ectoparasites, or search the incorrect host (if the ectoparasite is host-specific) or during the incorrect season. Regardless of familiarity level, trainings, whether administered in-person or online synchronously or asynchronously, should be considered to ensure standardized collection efforts and methods. By reducing the number of barriers that may preclude professionals with companion animal access from continuing to participate in a study, researchers are more likely to retain participants and have equally standardized quality of samples and sample collection efforts.

## 3. Personal Companion Animal Access

Another group of stakeholders that researchers can target for citizen science research is those who have personal companion animal access, which includes anyone of the general population who owns animals, including pets, livestock, or any domesticated or exotic animal. Similar to those who work with domestic animals, many of the studies working with this group have also focused on citizen-led efforts to collect data.

### 3.1. Surveillance and Data Collection Methodology

Like citizen scientists with professional access to animals, studies involving animal owners mostly focused on data submitted by citizen scientists in this group. One major difference between working with this group and those with professional companion animal access is that some of these studies did not specifically target pets and other domestic animals to collect samples. In other words, the study focused on opportunistic collections by people in or around the household and some participants happened to have animals and found ectoparasites on them or in adjacent areas. For these studies, the ectoparasites were either collected from the animal, found in the animal areas, or had blood meals indicating they fed on domestic animals [46,47,48,49].

Working with this stakeholder group, researchers utilized a variety of methods to passively sample ectoparasites, including using collection kits that were available through different avenues (available for pickup/drop off at researcher/Extension offices or sent to citizen scientists), submitting specimens to a research laboratory or institution, and submitting photos to a website or mobile device application. Unlike those who had occupations working with animals, only two studies that targeted animal owners utilized pre-made collection kits [15,24]. Instead, the majority of studies that involved animal owners advertised their studies online using a dedicated project website and/or outreach to recruit citizen scientists [18,37,46,47,48,49,50,51].

Even if vectors are not directly collected from animals, host associations between the vector and animal host can still be evaluated with a bloodmeal analysis of citizen-collected specimens. This is especially useful for vectors that are difficult to collect or transient in their feeding patterns, feeding for only a few minutes at a time before returning to their hibernaculum. For example, animal owners collected kissing bugs and noted that most triatomines were collected outside the home, often in dog kennels [48]. Subsequent bloodmeal analyses confirmed that the kissing bugs had previously fed on domestic pets such as dogs and cats [46,47]. Studies such as these leverage citizen scientist-collected data with molecular methods to determine the bionomics of arthropod vectors that are traditionally difficult to collect using active surveillance methods.

With nearly 85% of the adult population owning a smartphone, which have ever increasing camera capabilities [52], images submitted by animal owners is another option to collect data for vector surveillance [27,28]. These studies reinforced the idea that tick identifications via crowdsourced images can be used as a tool for risk assessment and tick monitoring on companion animals across large geographic scales as well as identifying hotspots for tick activity that put humans and their animals at risk for tick-borne diseases [27,28]. This provides a cost-effective choice for arthropod vector collection from animals by citizen scientists.

Similarly, the use of mobile phone apps can also be used for vector surveillance or identifying behaviors associated with arthropod bite risk. For example, the Tick App is a research-focused app that aims to understand human behavior related to tick exposure [30]. While mainly focused on collection and identification of ticks found on humans, the app included a questionnaire where users answered questions about lifestyle factors, such as pet ownership, and reported tick bites found on themselves and their pets. This information was then used to build an epidemiological profile for each user. Using this information, the authors identified human risk factors for tick exposure and validated the use of the app for research [30]. At this time, the use of images and mobile phone applications in North America for pet owners has been limited to tick surveillance.

### 3.2. Benefits

The benefits of working with animal owners are similar to those working with citizen scientists who have professional companion animal access, including monitoring trends of vectors and VBDs over extensive periods of time [36,37,38,49]. One unique benefit is that human risk for arthropod bites from their pets can be evaluated based on pet ownership [30] in addition to arthropod bite risk for companion animals [28]. Additionally, the number of targeted animal hosts can be increased since animal owners are not limited to the types of pets they can own, assuming that the pets fall within government regulations. Although we are not aware of any studies that targeted exotic animals and their owners, increasing the number of targeted animal hosts could broaden the scope of ectoparasites received from this population. Finally, depending on how data are collected, ectoparasites can be identified and/or tested for pathogens, which offers the opportunity to track range expansions of both the ectoparasite and the pathogens they harbor [15,27,50].

### 3.3. Challenges

Many of the possible challenges for those who have professional companion animal access also exist for those who have personal companion animal access. This includes finding and retaining citizen scientists who consistently check their animals for ectoparasites, including reporting and/or submitting specimens to researchers. Therefore, it is important to consider reminders and following up with citizen scientists regularly. Inconsistency in data collection is also possible due to inconsistent searching efforts, where animal owners may not be checking their animals regularly and opportunistically collecting arthropod vectors. A major benefit of working with animal owners in the general public is that there is the potential for a large sample size, but this may also introduce biases if a large percentage of samples comes from one region [53]. Analyses should therefore account for population size and geographic location when conducting studies on the prevalence of vectors and/or vector-borne pathogens using citizen scientist-collected data.

While smart phones and digital cameras are easily accessible for many populations, the use of photos as the only method to collect data points may limit some research objectives. This includes the uncoupling between vector presence and pathogen presence (unless photos are supplemented with physical specimens), limitations of smartphone cameras or photo output to accurately identify the vector, restrictions on the type of ectoparasite that can be identified via photographs (e.g., do not require microscopic examination), access to adequate cell or internet service, and less representation of participants from less affluent areas that may not be able to afford camera-enabled smart phones.

### 3.4. Stakeholder Considerations

Regardless of the challenges, animal owners can be an excellent resource to learn more about ectoparasites that affect companion animals. To work with this stakeholder group, researchers should consider the type of arthropods that the citizen scientists are required to collect. The arthropod must be large enough for the citizen scientists to see and it must be easily trapped or collected. Arthropods that require the use of a microscope or inaccessible tools to find and collect are not ideal for this citizen scientist group. The more difficult, expensive, or time-consuming it is to see and collect the arthropod, the greater the expected attrition rate of citizen scientists from the project. Providing collection supplies or requiring the use of items that are likely to be available in most households will make the research more accessible across many populations. Therefore, researchers should consider these factors when choosing the collection and reporting methods for citizen scientists. Likewise, depending on the ease of ectoparasite recognition, collection, and protocols to search the animal, trainings and instructions should be distributed to citizen scientists to ensure consistent data reporting.

## 4. Professional Wildlife Access

Citizen science can be especially effective when targeting experienced workers or experts in specific fields. Occupations that work closely with wildlife, such as animal control officers, game wardens, land managers, wildlife rehabilitators, and museum curators present unique opportunities to access wildlife. Unlike the studies that focused on people with professional or personal access to companion animals, researchers used either a passive surveillance technique or a more integrative approach, where researchers collaborated with workers that had professional access to wildlife to gather animals and/or arthropod vectors.

### 4.1. Surveillance and Data Collection Methodology

Some studies involving passive surveillance techniques provided sampling kits for citizen scientists with professional wildlife access [15,16,54,55,56]. Kits generally contained ethanol-filled vials for preserving collected specimens, data sheets for recording information about the collection event, mailers to return the collected arthropod vectors, and information on how to properly collect. Additional tools may be helpful depending on the collection method and arthropod. For example, Apperson et al. [54] provided forceps to aid collectors in removing attached ticks and Trout-Fryxell et al. [15] provided additional education resources, including tick information sheets, collection tutorial videos, and online publications through a website link included in the kits.

The remaining studies had the citizen scientists use their own collection containers for submissions [40,57,58,59,60,61,62]. Datasheets were not always provided to citizen scientists of this group, but some citizen scientists still reported relevant information about site of collection, species, etc. [40]. In general, datasheets or an online portal to submit information should be available for citizen scientists to enter information about their specimens for consistent data reporting.

The use of archived hosts to collect vectors has also provided additional information in a passive system. One study had museum curators collect, preserve, and provide ticks from archived wildlife hosts [56]. In this study, the authors worked with the Florida Fish and Wildlife Conservation Commission to collect ticks from wildlife that were archived between 2000–2014 [56]. By working with museum curators to collect ectoparasites from preserved hosts, researchers have the opportunity to track ectoparasites and identify historical host associations over extensive periods of time.

Thus far, entirely passive surveillance systems have worked well for those who have professional or personal companion animal access. In addition to passive surveillance of wildlife, researchers have opted for integrative methods to sample for ectoparasites in certain environments such as hunting check stations. In a few studies, researchers aided in the collection of ectoparasites at state-run game check stations [63,64]. In these studies, citizen scientists aided researchers directly on-site with ectoparasite collection. Other studies had those with professional wildlife access solely or assist with capturing and gathering target species, after which researchers collected the target ectoparasites [65,66,67,68].

Integrative collection techniques can be further extended outside of game check stations to deer meat processors. In states where hunting is popular but there are few-to-no check stations, animal processors can be an effective alternative for collection [22,69,70,71,72,73]. While deer processors can provide the animal carcasses to allow researchers to search for arthropods, this sampling often requires researchers on site to accomplish most of the ectoparasite collecting since workers at meat processors have limited time and are often busy during hunting season.

### 4.2. Benefits

Working with those who have professional access to wildlife can be simpler and more straightforward than other stakeholder groups. These citizen scientists are often already familiar with handling and researching wildlife, which can simplify trainings involving wildlife handling and identification as well as arthropod collection. This allows for passive surveillance with little direct oversight and perhaps more precise data collection efforts. For example, Corn et al. [59] relied on wildlife rehabilitators to submit ticks and accurately report on host species. Other citizen scientists may not have the specific expertise to differentiate between several exotic species without extensive training from researchers. Sample collection may also be easier if the citizen scientists are already regularly handling the animal of interest, which can facilitate animal processing and ectoparasite collection at a faster rate. For example, Ogden et al. [74] worked with bird observatories throughout Canada to investigate the role that migratory birds have in the distribution of *I. scapularis* and their associated pathogens. By working with professionals who regularly handle wildlife, researchers can increase their sample size and collection rate.

Because they usually work in a business or state agency, those with professional access to wildlife often have their contact information publicly available or can be contacted through official websites. Additionally, these state agencies and non-governmental organizations may have previously established relationships with universities and funding bodies that facilitate collaborations. Researchers can capitalize on previously established relationships between their universities/faculty/labs/organizations and build or continue vector surveillance programs with relative ease compared to other populations, with which an established relationship is required before starting the project [57,58].

Those with professional access to wildlife also frequently have access to recreational users or other groups that can help bolster the project and increase its reach. Hunter check stations, a frequently exploited professional wildlife accessor resource, are also frequently visited by successful hunters, who can assist with collecting animals or samples (see “Section 5” below). Relationships in these environments develop readily, opening another avenue of citizen science to willing researchers.

### 4.3. Challenges

Those with professional wildlife access are not equally distributed across all wildlife species. While opportunities such as hunter check stations are excellent resources for collecting ectoparasites, check stations are limited to game species and their presence, and the species checked varies by state. Big game, such as deer, elk, bear, and turkey, are the dominant game animals checked at check stations (Table S1). Unfortunately, physical hunter check stations have been phased out in many states in favor of electronic reporting, such as hunters reporting game online or via text message, although there has been a resurgence of mandatory physical deer checks due to concerns about chronic wasting disease.

Like the previous stakeholder groups, relying on personnel with professional access to wildlife can lead to some biases, depending on their spatial distribution throughout a county or state. If hunter check stations have been phased out, then this could result in unequal distributions of check stations throughout a county or state, leading to unequal representation of animals or trained personnel to check the animals for ectoparasites at the check stations. For example, if there is only one check station within a county that requires hunters to bring their animals, then a situation may arise where wildlife personnel may be inundated with their normal tasks to record required information and there may not be enough personnel or time to search animals for ectoparasites in a timely manner.

For states without mandatory check stations, game processors can provide access to wildlife. As mentioned previously, game processors themselves may not have the capacity to check for ectoparasites on the animals they process. Furthermore, hunters are not constrained in terms of bringing their animals to a processor that is near the harvest site and can instead choose to bring the animal to any processor, regardless of where it was harvested. By having unequal distributions of professionals with wildlife access to consistently search for ectoparasites on hunted animals, biases can arise and lead to erroneous data interpretations.

Animal control units are similarly limited as they are often focused around cities and are not present in all locations. Because of this, the animals they encounter are often domesticated species that are lost or feral (e.g., domestic cats and dogs), although synanthropic wild species, such as raccoons, skunks, opossums, and even coyotes, may be encountered as well. Two citizen science programs that worked with animal control units to collect ectoparasites both investigated Virginia opossum (*Didelphis virginiana*) hosts [66,68]. The animals in these studies are likely to represent a narrow set of environments in and around the city where animal control operates.

Wildlife rehabilitators may seem to be an occupational group that defies these limits. Depending on the facility, rehabilitators receive and interact with many animals of different taxa that are brought to them. A similar problem arises, however. The facility is only likely to receive injured animals that people have access to, which are more than likely many synanthropic species. In fact, most projects targeting wildlife rehabilitators were focused on ectoparasite collection from avian hosts [57,58,75], though one investigated reptile hosts [59].

### 4.4. Stakeholder Considerations

While professional wildlife accessors have access to wildlife by virtue of their day-to-day job, they often have their own agendas and tasks to accomplish on any given day. When utilized for research, these agendas should be considered. Citizen science programs that require long, onerous protocols are not ideal for occupational wildlife accessors since they are typically processing animals swiftly to reduce stress on the animal. These citizen scientists are best used for simple collections, especially those that align with their everyday tasks. For example, state officials often collect various age and health data (e.g., age, weight, body condition, etc.) from harvested game animals at check stations, so providing these data and ectoparasites to researchers would require little additional effort on their part. Furthermore, since they are quickly processing through many animals to satisfy their clientele, having professional wildlife accessors search for ectoparasites using an efficient systematic method (timed or by body section) may help them prioritize ectoparasite collection within their normal data collection or processing duties [22,73,76].

Because this group of people handles wild animals on a daily basis, they may already have access to collection materials if they are already collecting other animal-based data. This can save researchers’ time and funds dedicated to putting collection kits or materials together for professional wildlife handlers. Even with access to materials, ectoparasite collection should still be simple, quick, and straightforward to prevent delays in animal processing as part of the citizen scientists’ daily routine.

This citizen science group is large, capable, and extremely useful in many citizen science projects. They have the capacity to be fully trained in arthropod collection protocols prior to intervention from researchers and they encompass nearly all areas of wildlife health, management, and taxonomy.

## 5. Recreational Wildlife Access

Although community interaction with wildlife is not as extensive as in domestic animals and livestock, it still represents a large demographic in North America. Recreational wildlife users can be divided into two categories: consumptive, where the wildlife is killed or destroyed at the end of use, and non-consumptive, where the wildlife is unharmed. Annually, more than 100 million Americans engage in recreation oriented toward wildlife, whether that be consumptive or non-consumptive [11]. As urbanization and conservation efforts increase across the globe, human–wildlife interactions are increasing as well [77,78,79]. Thus, engaging these groups for citizen science research may be more successful and more fruitful than ever before.

### 5.1. Surveillance and Data Collection Methodology

The ways in which non-consumptive wildlife users interact with wildlife lends itself to passive collection methods. Most non-consumptive recreational wildlife activities are done year-round and independently. These activities include bird watching, feeding birds, photography, hiking, ecotourism, animal viewing, etc. Some recent studies have been able to leverage online citizen-submitted image databases, such as iNaturalist, eBird, BugGuide, Zooniverse, and various state herptile-focused websites, to gather data about the presence of host species, however, this has not been leveraged often with arthropods of veterinary importance. Three studies were found that use these tools for passive data collection [70,71,80]. Skvarla and Machtinger [70] and Skvarla et al. [71] used images of arthropods from online image databases to delineate deer ked geographic occurrence, while Putman et al. [80] took a different approach. Instead of identifying the arthropod independently of the host, the researchers analyzed tick burdens on alligator lizards (*Elgaria multicarinata*) by looking at image uploads of the lizards on iNaturalist [80]. Regardless of how the vectors are digitally identified, the use of already-existing databases comprising citizen scientist data provides an opportunity to conduct surveillance for arthropod vectors and host associations nationwide and beyond.

Most studies that targeted recreational wildlife users as citizen scientists targeted consumptive users specifically. These groups are active, usually state-registered, and create carcasses from which many ectoparasites can be easily collected. Similar to those with professional wildlife access, studies involving groups with recreational wildlife access used either a completely passive surveillance system or an integrative approach to data collection, where samples from recreational wildlife users were supplemented by researcher-led collection of ectoparasites or citizen scientists brought animals for researchers to sample. Researchers in some cases sent out collection kits and instructions and depended on hunters to collect, store, and return ectoparasites from their harvest [22,54,55,56,70]. These kits contained essential materials for preserving and returning samples to researchers, such as a datasheet, vials with ethanol solution, mailers to return the samples, and, in some cases, forceps and flea combs.

Hunters have historically been a major citizen science group contributing data to various studies involving the collection of ectoparasites from various game animals that may disperse or translocate vectors to new regions. Beyond ectoparasite collections, some hunters were asked to get more involved in data collection by removing and preserving animal hearts and delivering them to researchers to test the animals for VBDs [81]. Hunters in such states may be primed for organ collecting and may be more willing to collect additional game animal organs for things such as vector-borne pathogen screening. These passive techniques require significant input and effort by hunters as well as notable trust by researchers that survey techniques and effort are consistent and that data are reported accurately.

To help bridge the gap between accuracy, consistency, and researcher effort, many researchers used integrative approaches for vector collection events, where collection by research personnel supplemented citizen-submitted samples. Some relied on trained state-funded and/or volunteer personnel at hunter check stations to survey carcasses and collect ectoparasites [54,60] (also see “Section 4” above). Though these techniques allow for more control by the researchers, they also require more direct effort in training and recruiting personnel. These efforts may not always be necessary if a project does not require consistent and accurate survey methods.

When consistent survey methods are required, a different integrative approach may be more suitable, where citizens bring animals to researchers and the researchers search the animals for ectoparasites. This does not necessarily mean that the researcher finds and pursues the wildlife themselves, although researchers have reported harvesting animals for research in some cases [82]. Instead, most studies sampled game animals at hunter check stations, deer processors, and other high-volume recreational user areas to collect ectoparasites [60,63,76,83,84,85,86,87,88,89,90,91,92,93,94,95,96,97,98,99,100,101,102,103,104,105]. In this case, hunters would bring harvested animals to a central location and, then, as part of the animal checks or processing, researchers could search the animal for ectoparasites using an established systematic method. While this is not traditionally “citizen science” in the sense that citizens are not directly conducting the sampling, citizens are still involved in animal collection, which is a significant portion of on-host vector surveillance.

In terms of non-consumptive recreation groups, bird banders are a group that can be considered for citizen science projects given their historical contributions to vector and VBD research. In the 1930s, bird banders assisted with data collection by submitting ectoparasites found on birds to create a comprehensive list of ectoparasites affecting birds and bird health in the eastern U.S., thereby establishing the bird banding community as one of the first recreational citizen science groups involved in vector surveillance [106,107,108]. More recently, data collected by bird banders have also discovered range expansions of the Gulf Coast tick (*Amblyomma maculatum*) in Maryland [109], new host records of *I. scapularis* and associated pathogens from passerines in Canada [57,58,110,111], and hyperparasitism of mites and hippoboscids on birds [112].

### 5.2. Benefits

Recreational wildlife users are tremendously vast and varied. If a collection needs to be robust, widespread, and varied, this citizen science group can be essential to completing the project. Evans et al. [22] found that recreational wildlife users were able to submit more geospatially diverse samples compared to researcher-led sampling events. Additionally, the authors showed that once the citizen science group becomes familiar with an ectoparasite that was once unfamiliar, citizen scientists collected more samples than trained researchers in the following collection season [22].

Recreational wildlife users are also mostly members of the public. Though there are likely some demographic trends, recreational wildlife users can come from all aspects of society. This might be beneficial if the citizen science project is also centered around teaching and engaging the citizen scientists. Many citizen science projects have taken this approach in the past in other fields [113,114]. By having information about the arthropod vector(s) of interest on a website or included in outreach materials or collection kits, this particular stakeholder group can familiarize themselves with the targeted arthropods and become experts themselves during the collection process [22]. Opening lines of communication and actively engaging with the public through citizen science outreach has the potential to make the project even more impactful and long-lasting.

### 5.3. Challenges

Targeting consumptive wildlife users for citizen science is limited by the types of wildlife these users tend to harvest. Big game, including cervids and bears, dominate this sampling type because they are commonly hunted across the U.S. [11]. Big game species are also most likely to appear at check stations or processors, as opposed to small game animals, such as squirrels or rabbits, which do not usually need to be checked and are generally processed at home. Though some of the studies searched game birds for ectoparasites [56,65,67,115], these kinds of investigations were much less common and relatively specific when implemented, focusing on only one hunted clade or species. There are many species that can never be reached by hunters, anglers, and trappers since most migratory birds and many reptiles and amphibians are banned from harvest in the U.S. and Canada. Limiting the animal hosts that can possibly be included as consumptive wildlife will therefore limit the types of ectoparasites found on the host, especially if those arthropods are host-specific.

Sampling harvested animals can be ineffective for many vector species. Mosquitoes, most biting flies, and kissing bugs do not remain on the carcass after feeding. All but one study utilizing harvested animals investigated acarids, biting or chewing lice, and/or hippoboscids. The one exception is a study that indirectly analyzed host associations of kissing bugs and deer by detecting *Trypanosoma cruzi*, the causative agent of Chagas disease, in hunter-harvested deer hearts [81]. However, while detecting pathogens in animals can provide information regarding prevalence of pathogens in a wild population, it does not necessarily equate to vector surveillance or provide definitive evidence for host–vector interactions.

### 5.4. Stakeholder Considerations

Recreational wildlife users are usually not as specifically trained as some other citizen science groups. When conducting a project with this group, objectives and collection procedures should be explained clearly and specifically. Generally, those with an interest in certain animal species will be enthusiastic to assist, but they depend on the investigators to provide training and instruction at various levels of collection, such as animal handling or ectoparasite sampling. This could include physical and digital information sheets or media that can serve as a reference for the public throughout the duration of the project. These resources make it easier for researchers to answer questions and direct citizen scientists.

These stakeholders are often very enthusiastic about projects that they are involved in. Unlike occupationally involved citizen scientists, this group is often participating for free and on a voluntary basis because they might have a general interest in the wildlife populations they observe recreationally. This enthusiasm, when nurtured, can be very powerful and fruitful on both sides.

## 6. Gaps and Future Directions

Studies have previously focused on arthropod vectors collected from domestic pets, agricultural animals, and wildlife by harnessing citizen scientists’ participation, but there are clear gaps in terms of the vectors, animal hosts, and citizen scientist populations that could be represented in future studies. Many of the studies that were described in this review focused on vectors that had direct contact with the host for extended periods of time or stay with or around their hosts after feeding, such as hard ticks, fleas, deer keds, and kissing bugs. Interestingly, we did not find studies dedicated to passive citizen scientist collections of lice, which are often host-specific and stay with their animal hosts post-feeding. Instead, studies that collected lice from animals took an integrative approach, where citizen scientist submissions were supplemented by researcher collections or researchers actively searched for lice on animals collected by citizen scientists [65,69,108,115].

Citizen-based collections from animals of flying or transient insects, including mosquitoes, *Culicoides* biting midges, and filth and blow flies, were also noticeably missing from our broad literature search. This could be due to the challenges in collecting, transporting, and identifying these insects. Collections of these arthropods often rely on specific traps to collect samples without sacrificing the integrity of the specimens’ features required for identification. These traps are often expensive and require maintenance by trained professionals, which may present barriers to researchers or participants who do not have the funds to distribute or check the traps [116,117]. These arthropods are also difficult to photograph due to their small size and transient nature, feeding for only a few minutes and then leaving the host. While it is possible to photograph these arthropods and to have them identified using machine learning and artificial intelligence—as seen in Europe and Australia—these approaches have not been used in North America nor have they been used for surveillance from animals [7,31,32,33,34]. Therefore, future citizen science projects could evaluate the use of industry standard traps (e.g., BG traps, spot/sticky cards) that can be used by citizens to collect these arthropods around the home and from animals. Alternatively, perhaps there is a need for a new type of trap that can be easily maintained by citizens for the collection of flying and transient insects that are found around animal habitats. As technologies continue to improve, we might also see an increase in the use of digital data for zoophilic vector surveillance, whether the data are submitted directly to researchers or through online databases. While already used for some studies, the use of bloodmeal analyses to determine host associations may also be more common amongst citizen science programs that focus on transient vectors [46,47]. Overall, overcoming barriers to citizen science participation in collecting these arthropods will give involved parties the same opportunities and benefits we see when researchers work with citizen scientists to collect other arthropods from or around animals, such as ticks, kissing bugs, deer keds, and fleas.

Farmers and agricultural animals have also historically been underrepresented in citizen science projects involving the collection of arthropod vectors, including collections from cattle, poultry, sheep, goats, swine, and horses. While we included farmers in the Section 3, we did not find studies that specifically targeted this group for any ectoparasite collection. That is, studies were broadly requesting specimen collections from the general public, which includes farmers and their animals [15,24]. This was unexpected considering that farmers have previously provided large-scale spatial and temporal data in studies involving agricultural practices and farmland management [118,119,120], so farmers seem able and willing to collaborate with researchers. Regardless, farmers should be considered a valuable resource moving forward when it comes to collecting data and collaborating on projects involving vectors of animal and human interest. Data collected from agricultural animals by farmers could potentially provide surveillance data at fine spatial and temporal scales for vectors that affect agricultural animals or their owners, such as ticks, biting and filth flies, *Culicoides* biting midges, and lice. This will ultimately fill multiple research gaps regarding the surveillance of these livestock and poultry pests and the pathogens they carry.

Animal control units for wildlife and domestic animals also appear to be rarely utilized for passive collection of arthropod vectors. The studies that did work with them only investigated one of two ectoparasite hosts, the Virginia opossum and feral cats [20,66,68]. The capture and trapping expertise of animal control units, as well as their focus around cities and other populated areas, make them an effective resource for ectoparasite collection from various animal species. Research targeting synanthropic hosts or One Health concepts might benefit from collaborating with these departments as research specifically focusing on opossum hosts and feral cats have proven successful with these communities.

Wildlife rehabilitators were a similar resource for passive collection that may be under-exploited. Wildlife rehabilitation centers generally host a variety of wildlife from many taxa. The wild animals are generally sick or injured, and so are likely to be more vulnerable to ectoparasites. Despite this, only four studies engaged with this community, three of which focused on avian hosts [57,58,59,75]. Future research should, therefore, focus on this community for passive collection of a large variety of ectoparasites or generalist ectoparasites across many animal hosts.

While gaps in the literature exist for citizen science projects focused on vector surveillance of animals, future studies should pursue these understudied topics and populations. Doing so will advance vector research and knowledge of arthropod vectors and the pathogens they carry in a changing landscape.

## 7. Conclusions

With increases in urbanization, climate change, and other anthropogenic events, cases of VBDs in humans and animals will continue to rise [1,121,122]. Because animals can serve as reservoirs for zoonotic diseases that can spillover into human populations, a One Health approach involving vector and VBD surveillance in animal populations is vital for human and animal health. Given that some methods of vector surveillance can be labor-intensive, expensive, time-consuming, or difficult to conduct for health departments or agencies alone, citizen scientists can fill this gap for vector surveillance and monitoring [123]. Because many previously published reviews focused on citizen science-based collections of arthropod vectors of humans [6,45], this review compiled contemporary studies focused on citizen science projects that collected arthropods from animals. This review highlights the possible stakeholder groups that can be targeted as potential citizen scientists to collaborate on projects involving vector and VBD surveillance in North America. We also identified the types of collection methods, benefits, challenges, and considerations when working with these stakeholders.

**Figure 1:**
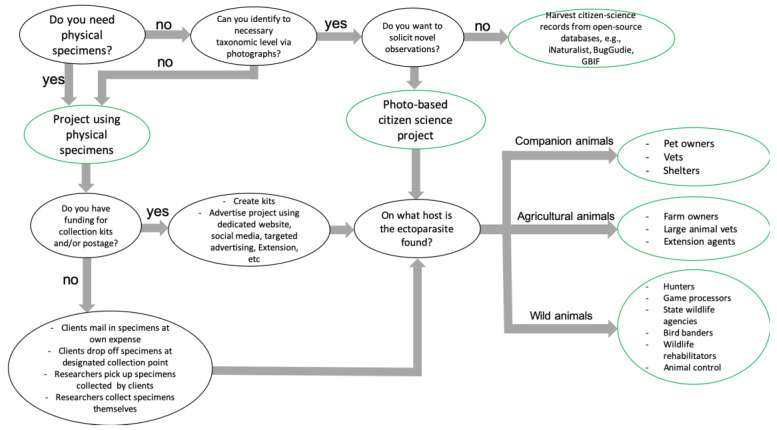
A flowchart of considerations and questions related to ectoparasite collection, animal populations, and target audiences while planning a citizen science program focused on ectoparasite sampling from animal hosts.

While there are differences in working with various groups of citizen scientists, similar patterns begin to emerge regarding considerations when collaborating with specific groups. When working with citizen scientists, researchers should consider the following information related to the ectoparasite, animal host, and target populations when designing a citizen science program (a flowchart of considerations can be found in Figure 1):

• Target audience;
• Type of arthropod(s) to be collected;
• Animal population(s) to sample;
• Ease of collection and preservation methods, including time and personnel required to collect;
• Cost of collection and preservation methods (for the researchers and participants);
• Geographic location and population density; and
• Training and outreach required to recruit and retain citizen scientists in the research.

Researchers can use these considerations to decide if a completely passive or integrative approach would be more appropriate or feasible and the types of data that can be collected with citizen scientist efforts. By contemplating these considerations, researchers can expect success in their citizen science program beyond just data and research, but they can also expect success in community engagement, trust, and communication amongst all parties, leading to longtime partnerships and collaborations.

Whether citizen science works for a project regime or not, more effort should be made to involve the public in scientific research. It is often thought that science informs the community, but observation informs science. Community involvement with science can help focus and transform research projects to be more effective and sometimes more novel. In this sense, science should be a cyclical two-way conversation between the researchers and the communities they inform, where questions and answers are developed and refined by both parties. Working together towards the goal of One Health, researchers and citizen scientists can learn from one another and contribute to science that will improve human and animal health.

## Data Availability

Not applicable.

## Supplementary Materials

The following are available online at https://www.mdpi.com/article/10.3390/insects13060492/s1, Table S1: Game check stations by state.
